# Supplementary material for: Unveiling the additive-assisted oriented growth of perovskite crystallite for high performance light-emitting diodes
Source: Nat Commun. 2021 Aug 23;12:5081. doi: 10.1038/s41467-021-25407-8 (PMC8382739; doi:10.1038/s41467-021-25407-8)
Supplement: Supplementary file 1 — Supplementary Information [file 41467_2021_25407_MOESM1_ESM.pdf]

Supplementary Information

Unveiling the Additive-Assisted Oriented Growth of Perovskite Crystallite for High Performance Light-Emitting Diodes

Lin Zhu[1]†, Hui Cao[1]†, Chen Xue[2]†, Hao Zhang[1], Minchao Qin[3], Jie Wang[1], Kaichuan Wen[1], Zewu Fu[1], Tao Jiang[1], Lei Xu[1], Ya Zhang[1], Yu Cao[1,2], Cailing Tu[1], Ju Zhang[1], Dawei Liu[1], Guangbin Zhang[1], Decheng Kong[1], Ning Fan[1], Gongqiang Li[1], Chang Yi[1], Qiming Peng[1], Jin Chang[1], Xinhui Lu[3], Nana Wang[1]*, Wei Huang[1,2]*, Jianpu Wang[1]*

[1]Key Laboratory of Flexible Electronics (KLOFE) & Institute of Advanced Materials (IAM), Jiangsu National Synergetic Innovation Center for Advanced Materials (SICAM), Nanjing Tech University (NanjingTech), 30 South Puzhu Road, Nanjing 211816, China.

[2]Shaanxi Institute of Flexible Electronics (SIFE), Northwestern Polytechnical University (NPU), 127 West Youyi Road, Xi'an 710072, China.

[3]Department of Physics, The Chinese University of Hong Kong, Shatin 999077, Hong Kong.

Corresponding authors: iamnnwang@njtech.edu.cn, iamwhuang@nwpu.edu.cn, iamjpwang@njtech.edu.cn.

†These authors contributed equally to this work.

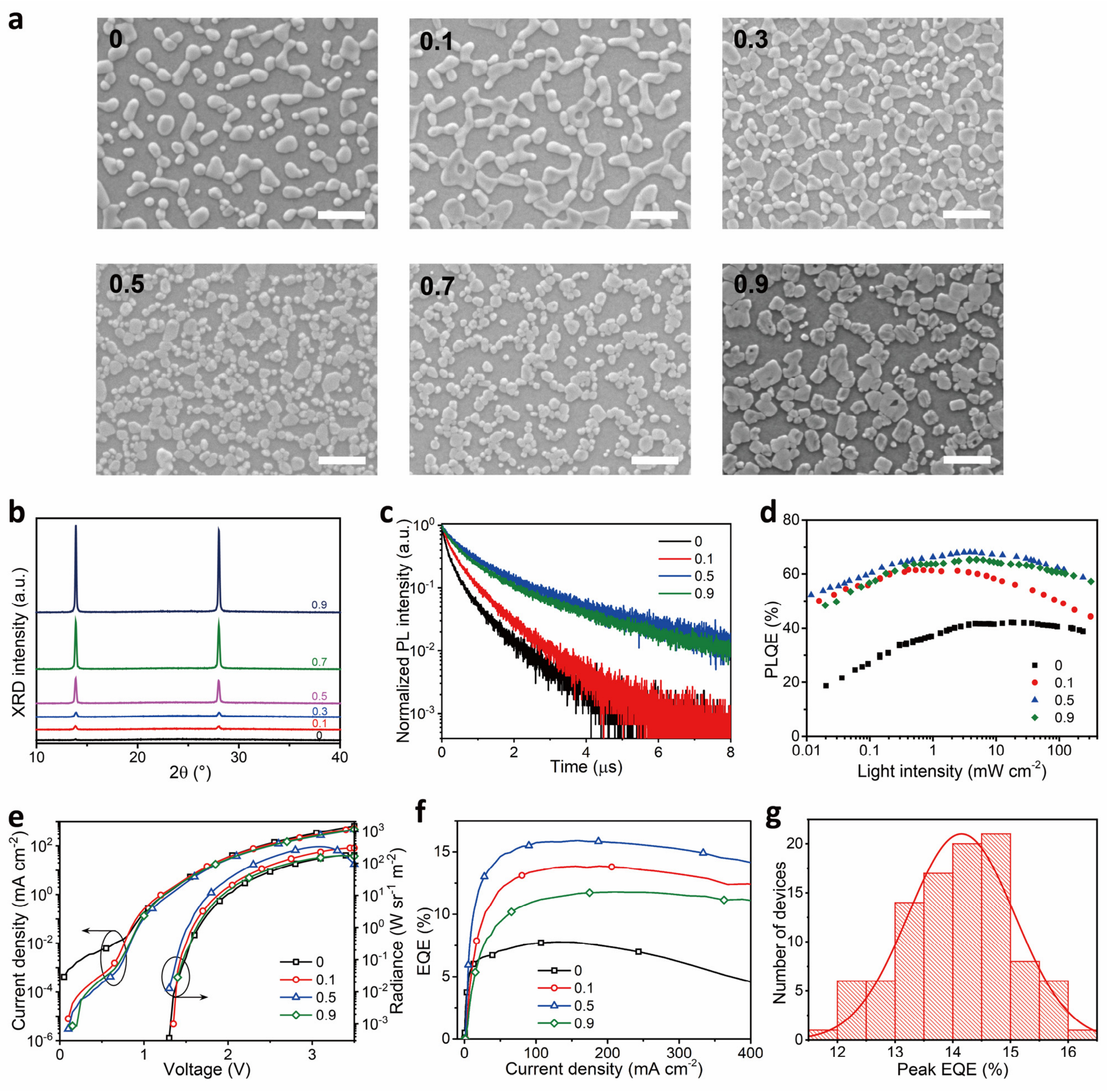

Supplementary Figure 1. Perovskite films and LEDs with various PAM amounts. a, SEM images (Scale bar: 1 μm). **b**, XRD data. By using Scherrer's equation ($D = K\lambda/(\beta\cos\theta)$, where D is the size of crystallite, k=0.89, λ=1.5406 Å, β is the full width at half maximum intensity of the peak, θ is the Bragg angle), the crystallite sizes of perovskites with various PAM ratios are calculated as 23, 27, 28, 42, 44, 60 nm, respectively. **c**, Time-resolved PL decay transients (excitation intensity of 7 nJ cm^{-2}). **d**, Excitation-intensity dependent PLQE. **e**, Current density and radiance versus voltage. **f**, Dependence of EQE on current density. **g**, Histogram of peak EQEs of 100 devices with 0.5 ratio PAM. The devices show an EQE of 14.2±0.9% with an error corresponding to the standard deviation.

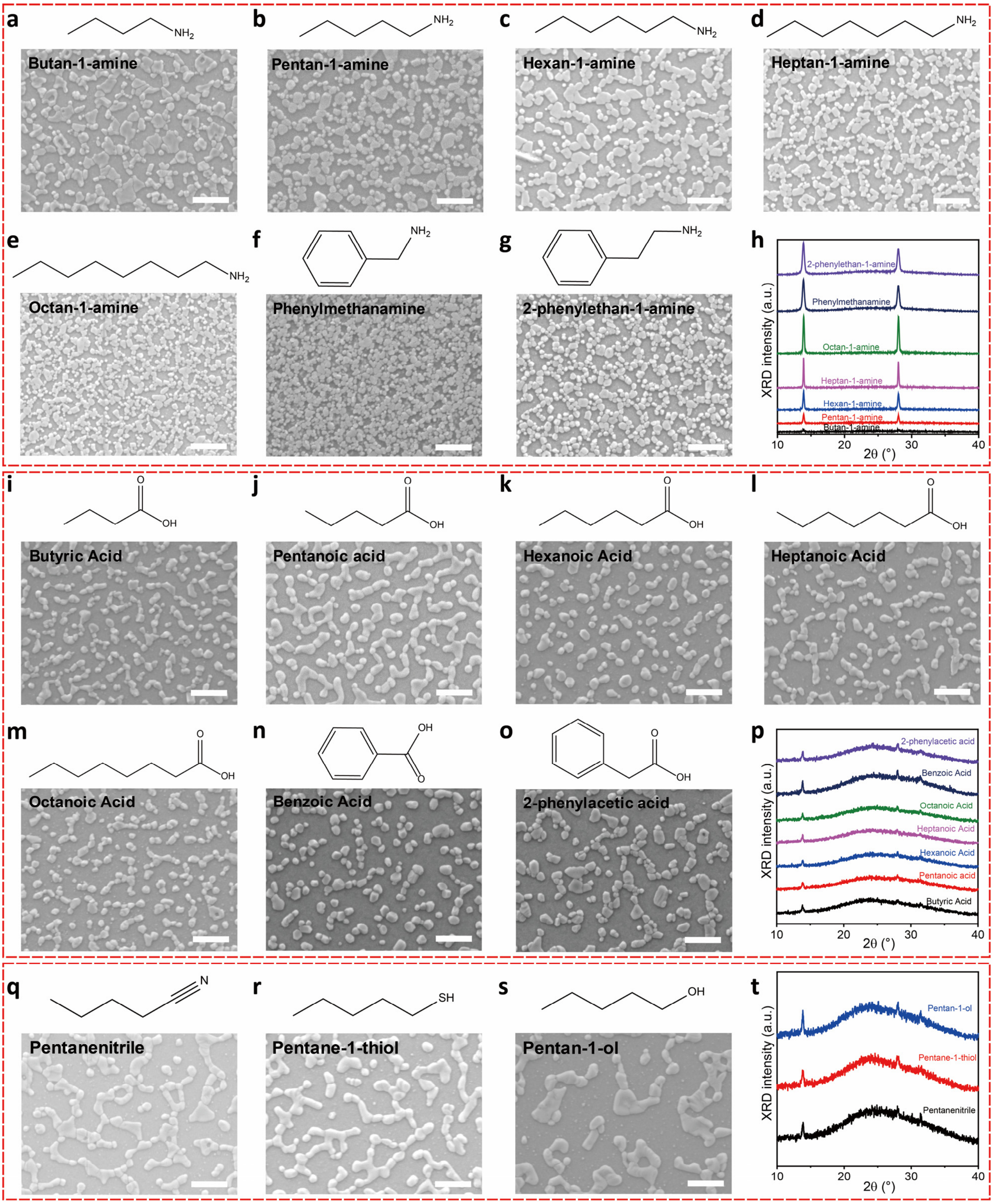

Supplementary Figure 2. Morphology and crystallinity of $FAPbI_3$ perovskites with various additives. The chemical structures of additives are shown on top of SEM images. **a-g**, SEM images of perovskites with amine-group additives. Scale bar: 1 μm. Butan-1-amine (a), Pentan-1-amine (b), Hexan-1-amine (c), Heptan-1-amine (d), Octan-1-amine (e), Phenylmethanamine (f), 2-phenylethan-1-amine (g). **h**, XRD patterns of perovskites with amine-group additives, which show no peaks for 2D

phase. **i-o**, SEM images of perovskites with carboxyl-group additives. Scale bar: 1 μm. Butyric acid (i), Pentanoic acid (j), Hexanoic acid (k), Heptanoic acid (l), Octanoic acid (m), Benzoic acid (n), 2-phenylacetic acid (o). **p**, XRD patterns of perovskites with carboxyl-group additives. **q-s**, SEM images of perovskites with Pentanenitrile (q), Pentane-1-thiol (r) and Pentan-1-ol (s) as additives. Scale bar: 1 μm. **t**, XRD patterns of perovskites with Pentanenitrile, Pentane-1-thiol and Pentan-1-ol as additives.

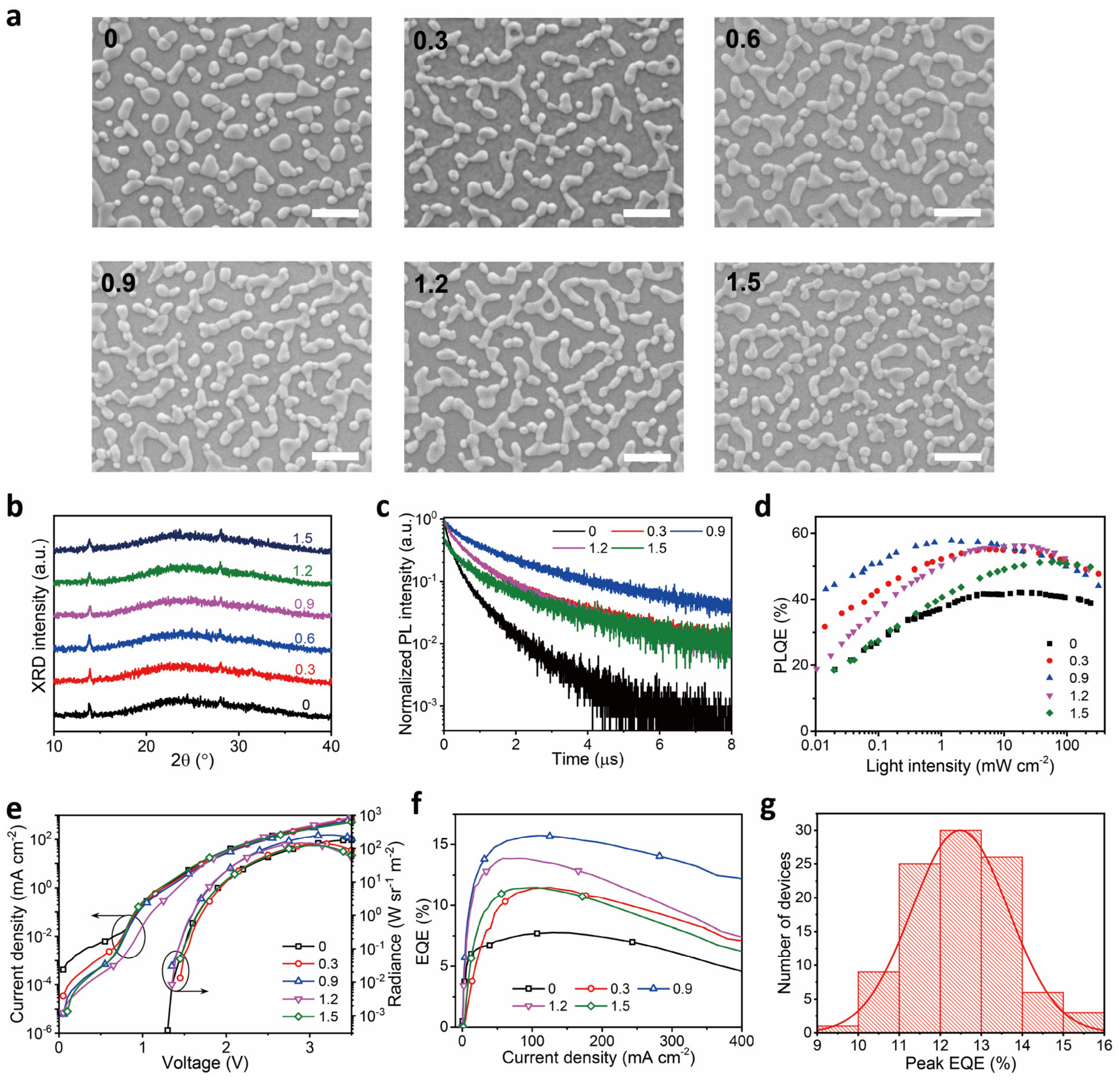

Supplementary Figure 3. Perovskite films and LEDs with various PAC amounts. a, SEM images (Scale bar: 1 μm). **b**, XRD data. **c**, Time-resolved PL decay transients (excitation intensity of 7 nJ cm^{-2}). **d**, Excitation-intensity dependent PLQE. **e**, Current density and radiance versus voltage. **f**, Dependence of EQE on current density. **g**, Histogram of peak EQEs of 100 devices with 0.9 ratio PAC. The devices show an average EQE of 12.5±1.2% with an error corresponding to the standard deviation.

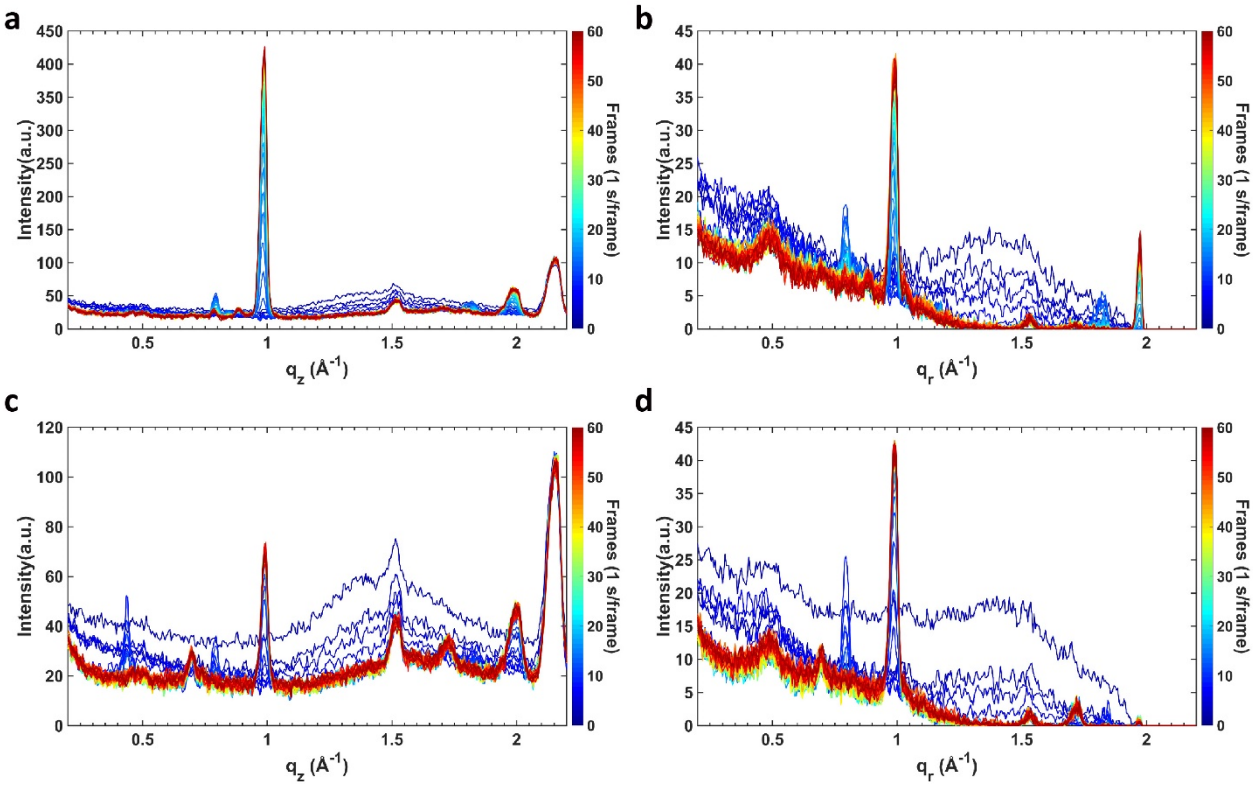

Supplementary Figure 4. In-situ GIWAXS intensity profiles of $FAPbI_3$ perovskites with PAM and PAC. a-b, The GIWAXS intensity profiles along the q_z (a) and q_r (b) directions for the PAM-based perovskite. **c-d**, The GIWAXS intensity profiles along the q_z (c) and q_r (d) directions for the PAC-based perovskite.

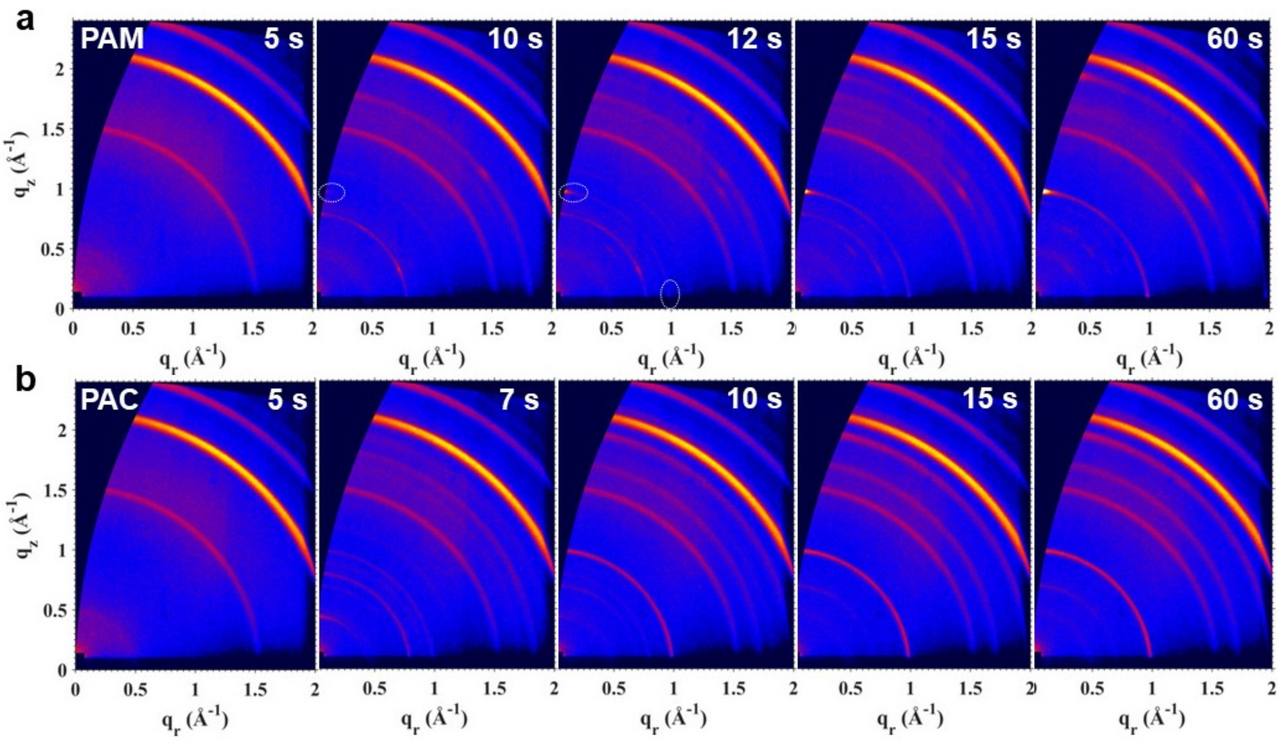

Supplementary Figure 5. GIWAXS pattern evolutions during spin-coating process. a-b, Representative 2D GIWAXS patterns versus spinning time of PAM-based (a) and PAC-based (b) perovskites.

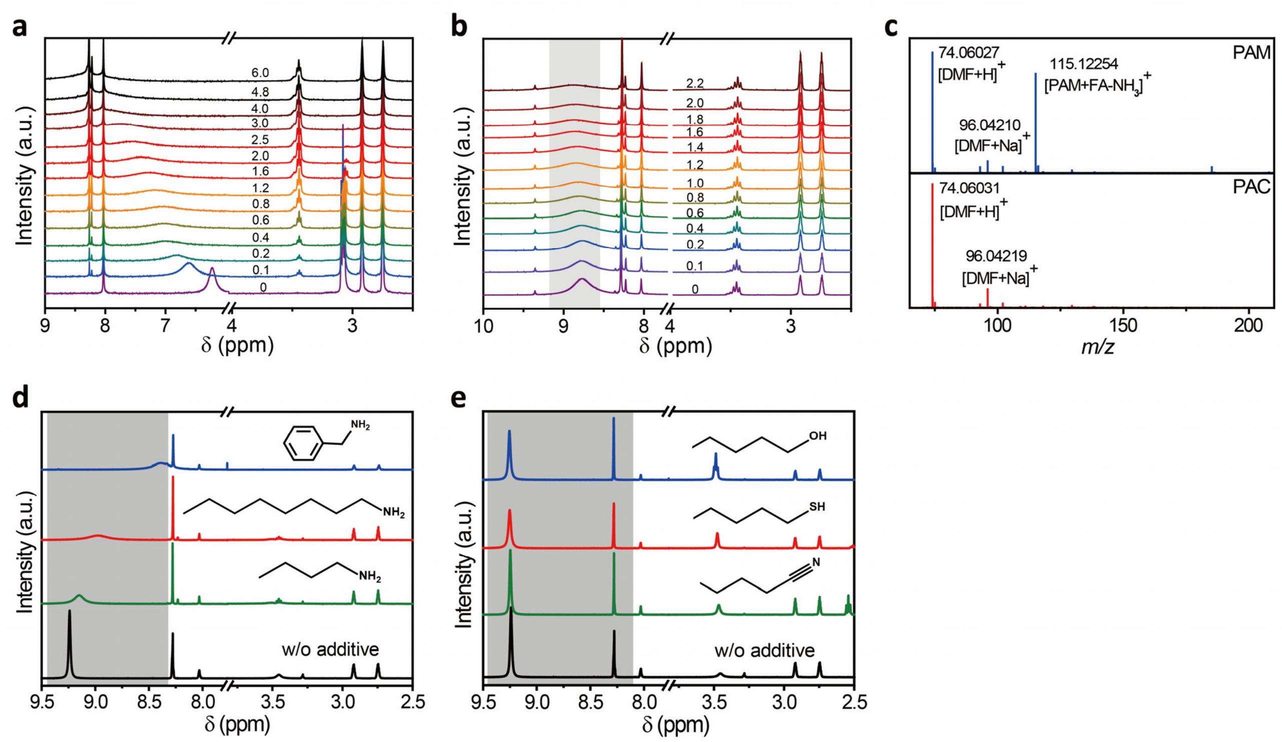

Supplementary Figure 6. ^{1}H NMR titration experiments (DMF-*d*7, 500 MHz) and mass spectra. **a**, Changes in ^{1}H NMR spectra of PAM•PbI_2 (0.078 M) on addition of FAI (0-6.0 equiv) in DMF-*d*7. **b**, Changes in ^{1}H NMR spectra of PAM•FAI (0.078 M) on addition of PbI_2 (0-2.2 equiv) in DMF-*d*7. **c**, ESI-TOF-MS spectra of PAM-based and PAC-based perovskite solution in DMF. **d-e,** ^{1}H NMR spectra of FAI solutions with amine additives (d) and other functional-group additives (e).

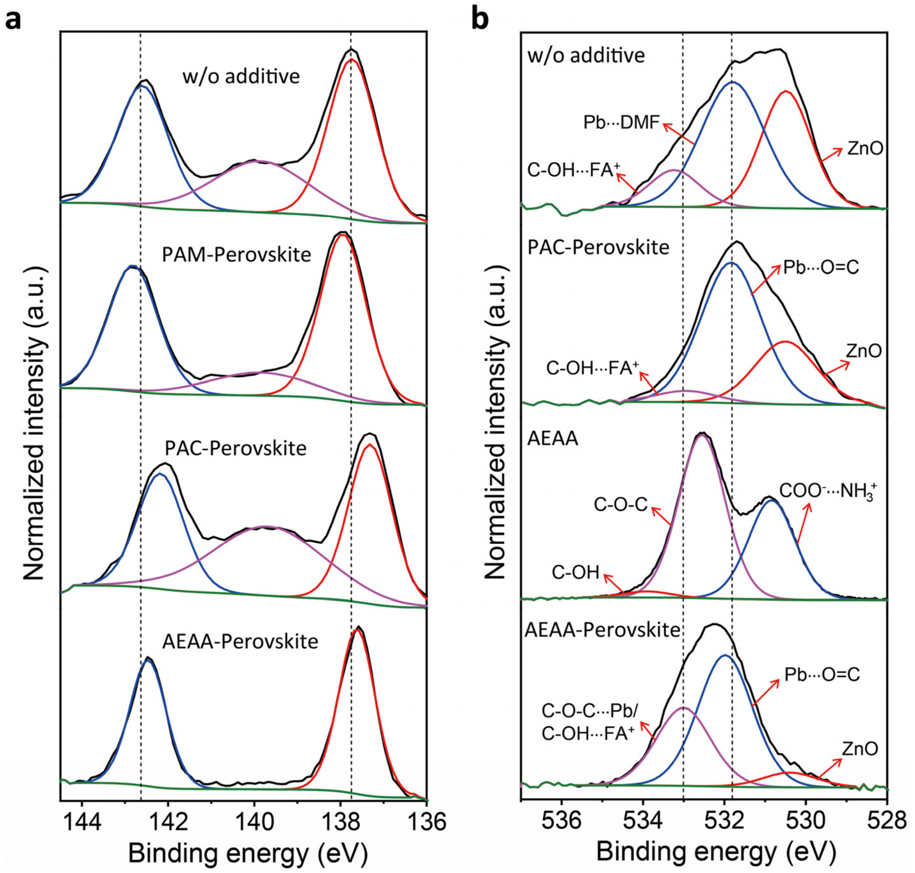

Supplementary Figure 7. XPS spectra of the control, PAM, PAC and AEAA-based perovskite films. a, Pb 4*f* core-level spectra. The pink peak is the Zn 3*s* signal from the ZnO/PEIE substrate. In the PAM based perovskite, the signal of Pb 4*f* state moves to higher binding energy, which is due to the effect of amine group in determining the crystal growth process, leading to perovskite film with fewer defect. **b**, O 1*s* core-level spectra. The peaks at 530.0 and 533.2 eV of the control sample come from the ZnO/PEIE substrate, and the peak at 531.8 eV is assigned to the DMF solvent. The C=O peak of the PAC-based perovskite film moves to 531.8 eV compared with the 531.3 eV of intrinsic C=O peak[1] and shows enhanced intensity compared with the ZnO substrate. Combining with the shift of peak position in the Pb 4*f* spectrum of PAC-based perovskite film to the lower binding energy region, this indicates that the carbonyl group can coordinate with the unsaturation Pb, passivating the iodine defect. The zwitterion AEAA has three species of oxygen atoms, which are C-OH located at 534.0 eV, C-O-C at 532.6 eV and COO^- at 530.8 eV. Similar as PAC-based perovskite, the AEAA-based perovskite film shows shift of peak position in the Pb 4*f* spectrum to the lower binding energy, along with the move of C=O peak to higher binding energy, which suggests the coordination between carbonyl group and unsaturation Pb. Moreover, the intensity of peak at 533.0 eV increases, which may originate from the interaction of C-O-C species with FA^+ or unsaturation Pb.

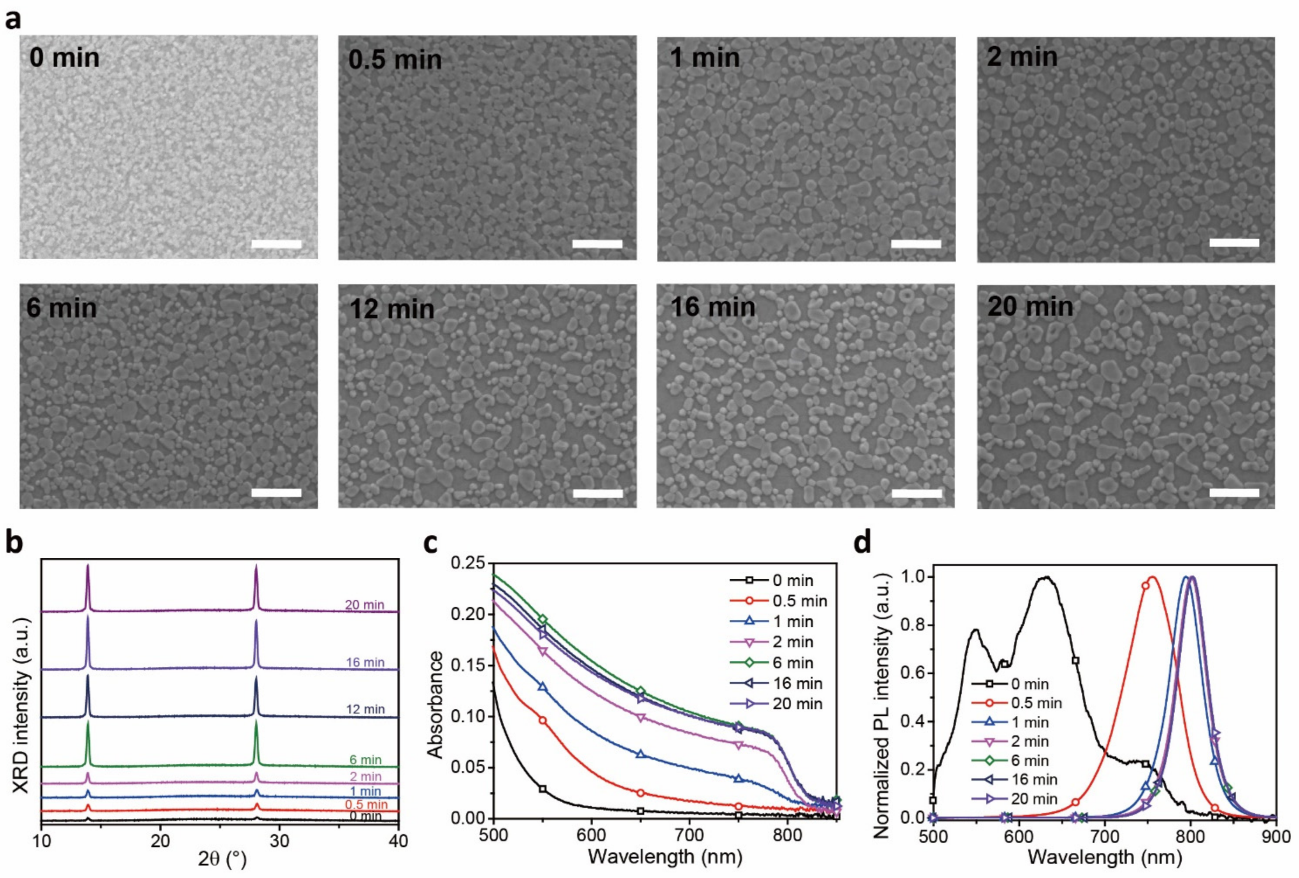

Supplementary Figure 8. Characterizations of PAM-based perovskite films with various annealing time. a, SEM images. Scale bar: 1 μm. **b**, XRD data. **c**, Absorbance spectra. **d**, PL spectra.

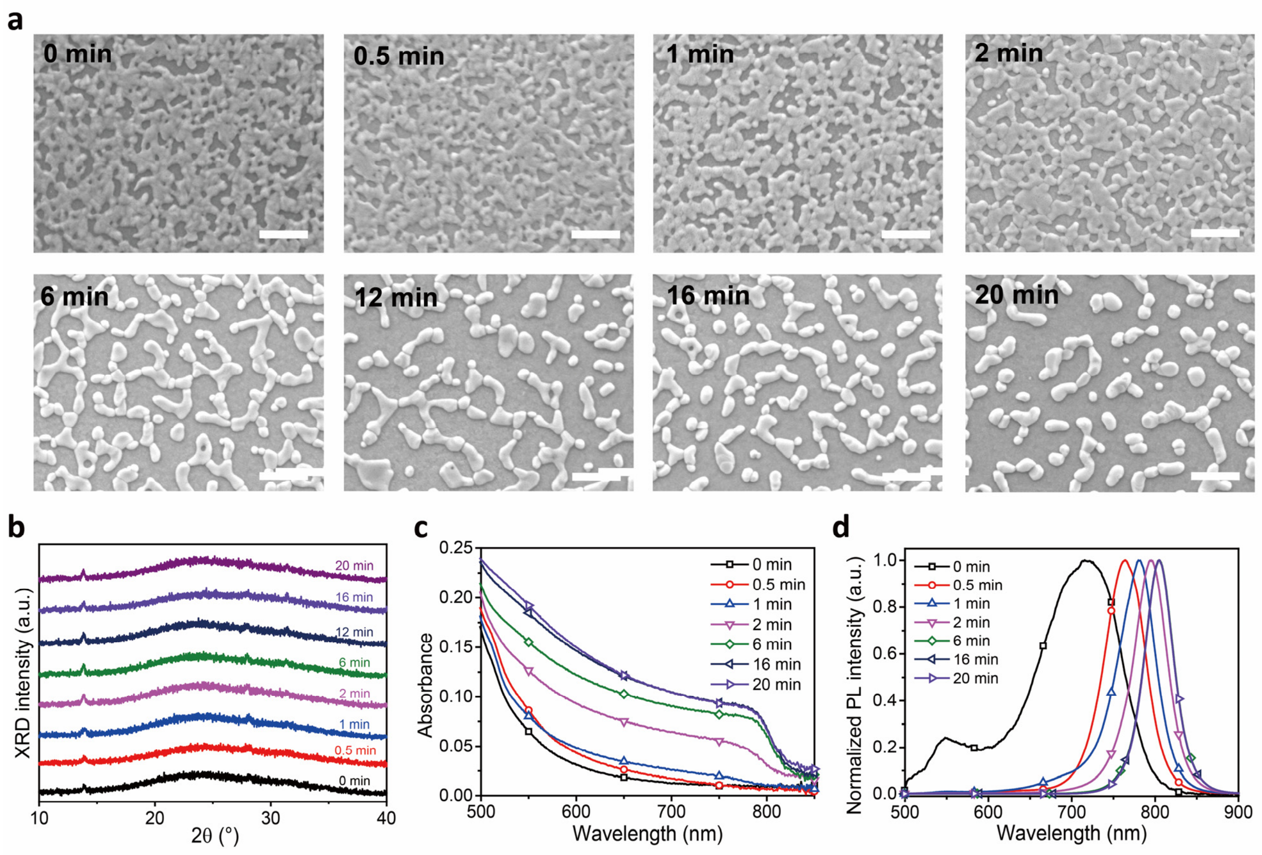

Supplementary Figure 9. Characterizations of PAC-based perovskite films with various annealing time. a, SEM images. Scale bar: 1 μm. **b**, XRD data. **c**, Absorbance spectra. **d**, PL spectra.

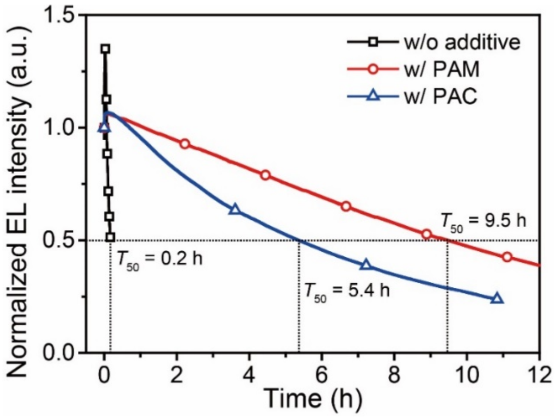

Supplementary Figure 10. Stability of LEDs measured at a constant current density of 100 mA cm^{-2}.

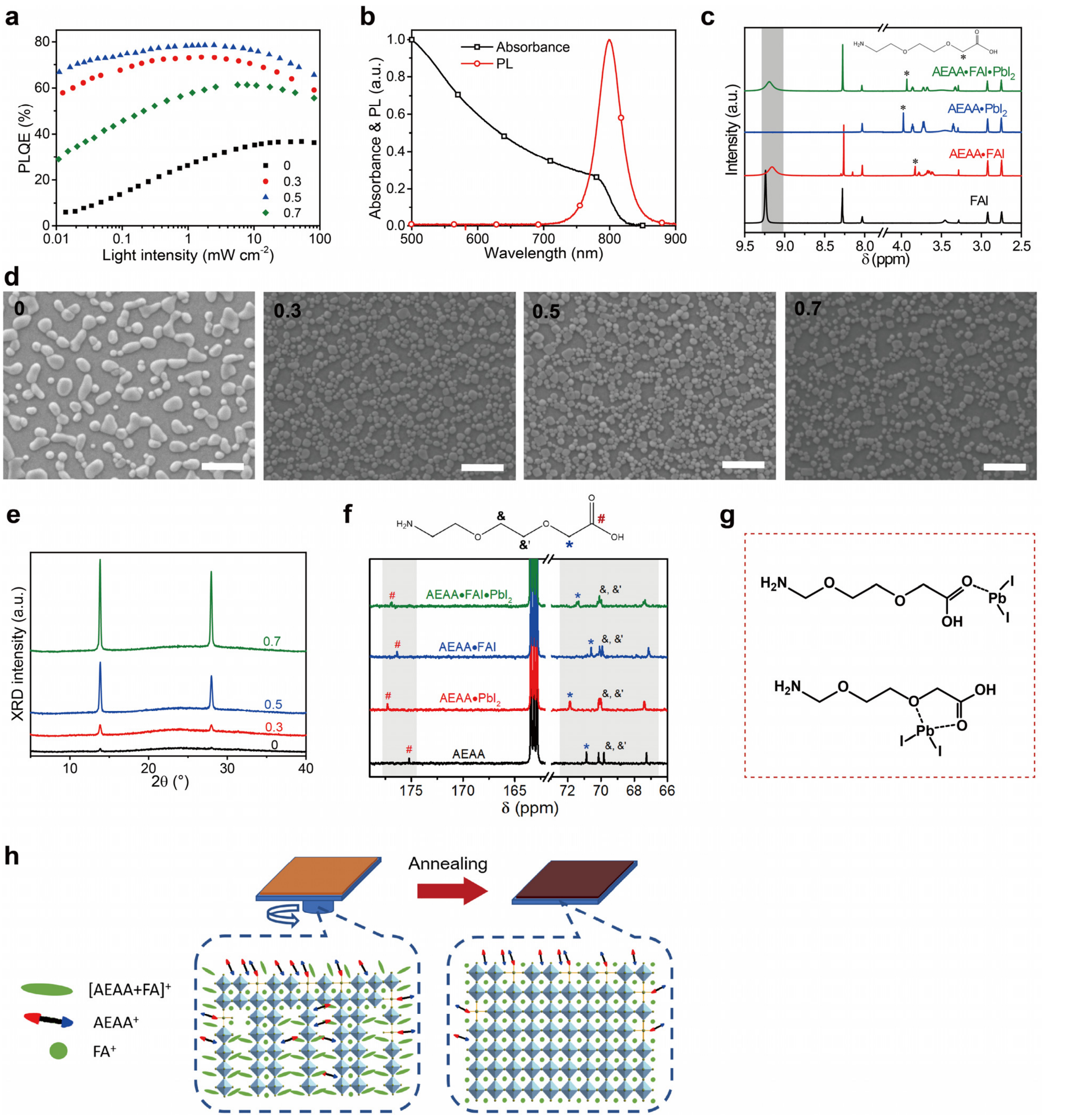

Supplementary Figure 11. Characteristics of AEAA-based perovskite films. a, Excitation-intensity dependent PLQE. **b**, Absorption and PL spectra. **c**, ^{1}H NMR spectra (DMF-d7, 500 MHz) of AEAA-based perovskite precursor solution. **d**, SEM images (Scale bar: 1 μm). **e**, XRD data. **f**, ^{13}C NMR spectra ($V_{d7\text{-}DMF}$:V_{D2O}=9:1, 400 MHz) of AEAA-based perovskite precursor solution. It shows significantly changed signals of AEAA at 175.21 (**C**OOH), 70.88 (O-**C**H_2-COOH), 70.14 and 69.83 ppm (O-**C**H_2-**C**H_2-O) after the addition of PbI_2 or FAI, which confirms that the COOH and C-O-C groups of AEAA can interact with both PbI_2 and FAI. **g**, Schematic illustration of coordination mode between carboxyl, ether groups and unsaturation Pb. The carboxyl group can directly coordinate

with unsaturation Pb or interact with unsaturation Pb through the assistance of ether group by forming a chelate ring. **h**, Schematic illustration of growth pathways of $FAPbI_3$ perovskites with AEAA.

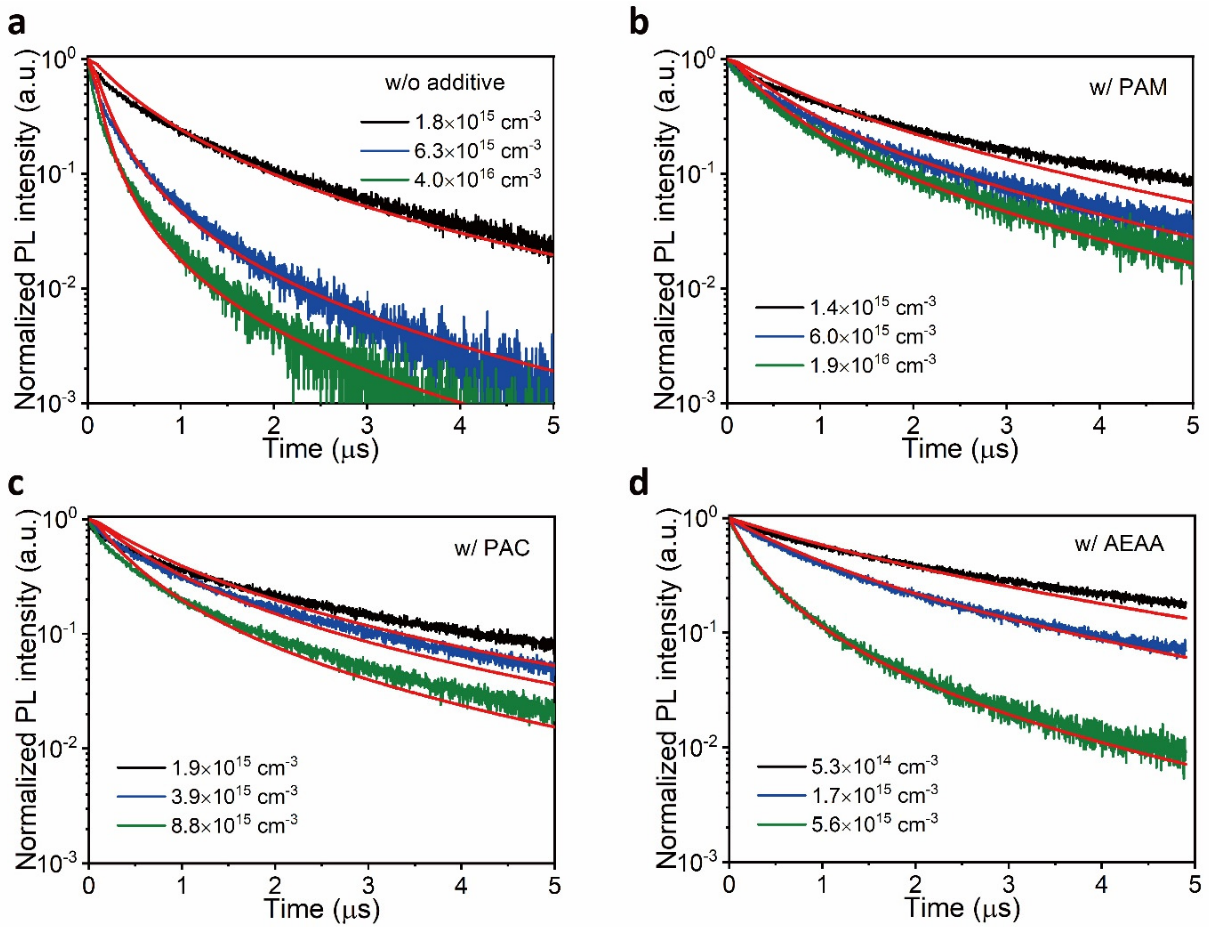

Supplementary Figure 12. Time-resolved PL decay transients of perovskite films under different excitation intensities. Red lines are fits from the generic kinetic model[2]. **a**, Perovskite film without additive. **b**, PAM-based perovskite film. **c**, PAC-based perovskite film. **d**, AEAA-based perovskite film. The trap densities of perovskite films without or with PAM, PAC and AEAA additives are 7.5×10^{15}, 2.0×10^{14}, 2.4×10^{14} and 9.1×10^{12} cm^{-3}, respectively. The PL lifetime of corresponding films at a carrier density of around 2×10^{15} cm^{-3} are 0.6, 1.1, 1.0 and 1.1 μs, respectively. The PL lifetime is defined as the PL intensity drops to 1/*e* of its initial intensity.

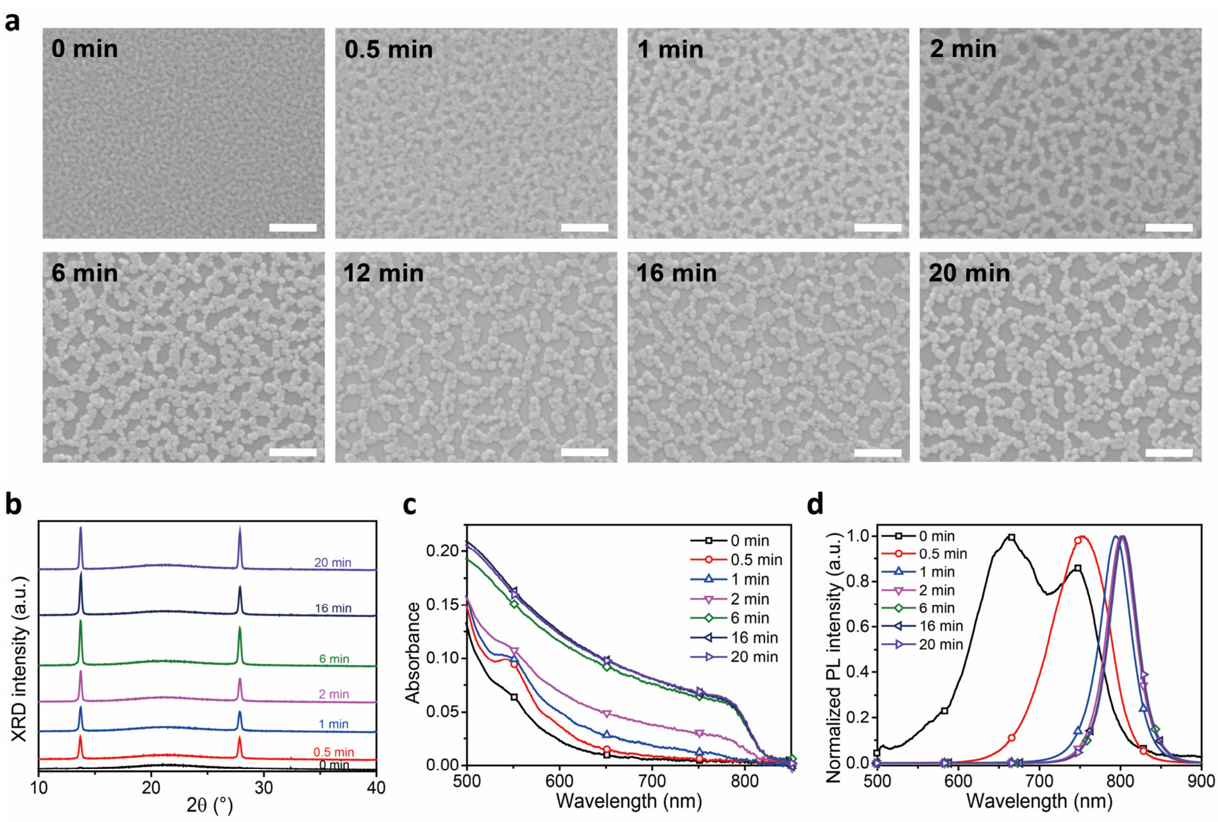

Supplementary Figure 13. Characterizations of AEAA-based perovskite films with various annealing time. a, SEM images. Scale bar: 1 μm. **b**, XRD data. **c**, Absorbance spectra. **d**, PL spectra.

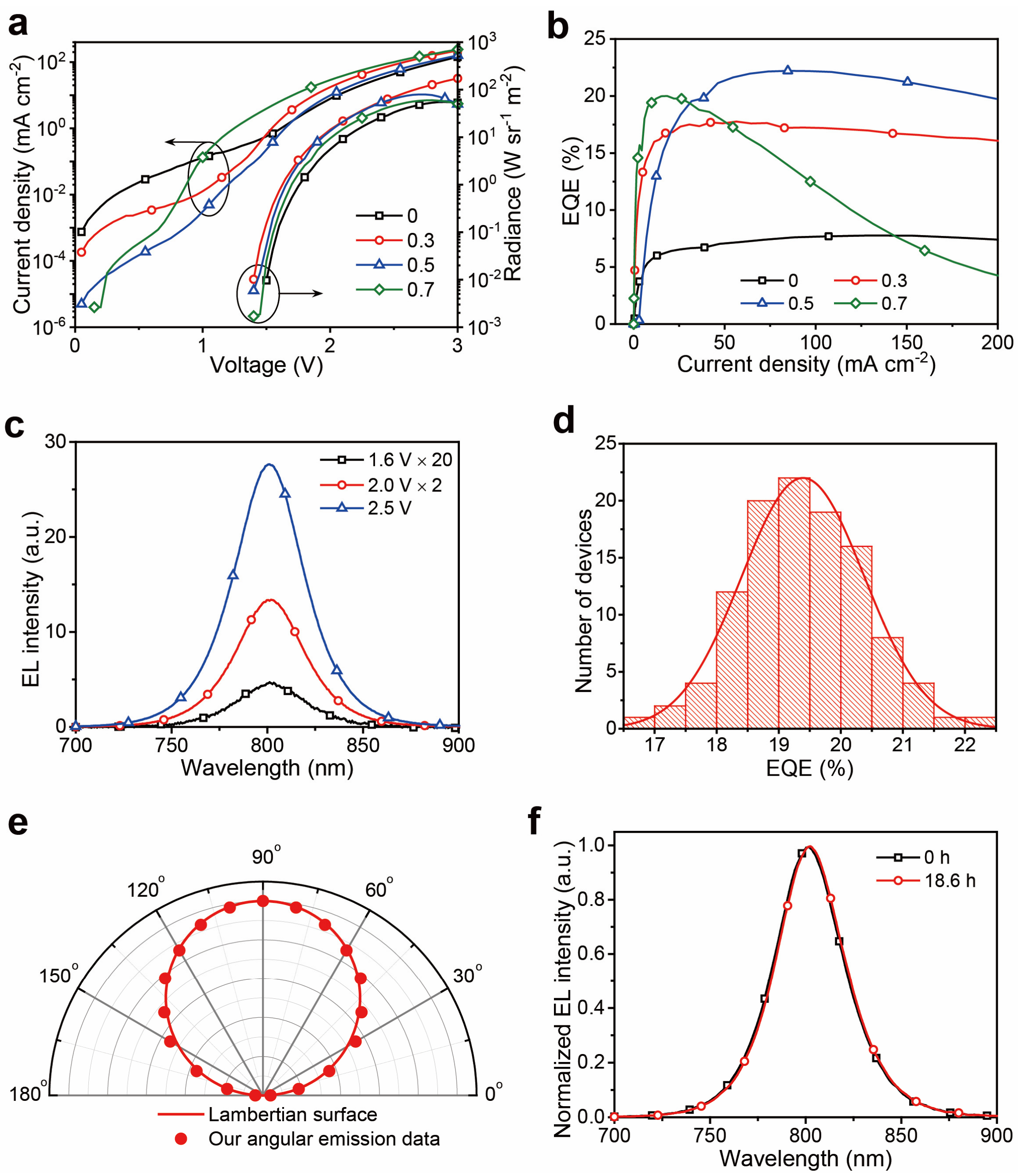

Supplementary Figure 14. Characteristics of AEAA-based perovskite devices. **a**, Current density and radiance versus voltage. **b**, Dependence of EQE on current density. **c**, EL spectra of the champion device upon various biases. **d**, Histogram of peak EQEs from 110 devices (0.5-ratio AEAA). The devices show an average EQE of 19.4±1.0% with an error corresponding to the standard deviation. **e**, Angular dependent profile of the emission of AEAA perovskite LEDs, which follows a Lambertian profile. **f**, EL spectra of the 0.5-ratio AEAA-based device upon long-term bias. The shape of the EL spectra remains unchanged.

Supplementary Table 1 Comparison of our perovskite LED with other high-performance NIR devices.

Emission layer	EL peak (nm)	Peak EQE (%)	T_{50} (h)	Current density for stability measurement (mA cm^{-2})	Reference
$FAPbI_3$ (AEAA as additive)	800	22.2	19	100	This work
$FAPbI_3$ (ODEA as additive)	800	21.6	25	20	[3]
$FAPbI_3$ (5AVA as additive)	803	20.7	20	100	[4]
NMA-$FAPbI_3$ (poly-HEMA as additive)	~800	20.1	46	0.1	[5]
$FAPbI_3$ (pimelic acid as additive)	802	18.6	682	20	[6]
$FA_{0.83}Cs_{0.17}PbI_3$ (PPAI as surface passivation molecule)	789	17.5	130	100	[7]

Supplementary References:

1. Stevens, J. S. *et al.* Quantitative analysis of complex amino acids and RGD peptides by X-ray photoelectron spectroscopy (XPS). *Surf. Interface Anal.* **45**, 1238–1246 (2013).
2. Stranks, S. D. *et al.* Recombination kinetics in organic-inorganic perovskites: excitons, free charge, and subgap states. *Phys. Rev. Appl.* **2**, 034007 (2014).
3. Xu, W. *et al.* Rational molecular passivation for high-performance perovskite light-emitting diodes. *Nat. Photonics* **13**, 418–424 (2019).
4. Cao, Y. *et al.* Perovskite light-emitting diodes based on spontaneously formed submicrometre-scale structures. *Nature* **562**, 249–253 (2018).
5. Zhao, B. *et al.* High-efficiency perovskite–polymer bulk heterostructure light-emitting diodes. *Nat. Photonics* **12**, 783–789 (2018).
6. Kuang, C. *et al.* Critical role of additive-induced molecular interaction on the operational stability of perovskite light-emitting diodes. *Joule* **5**, 618–630 (2021).
7. Guo, Y. *et al.* Phenylalkylammonium passivation enables perovskite light emitting diodes with record high-radiance operational lifetime: the chain length matters. *Nat. Commun.* **12**, 644 (2021).
